# Supplementary material for: Patterns of Intron Gain and Loss in Fungi
Source: PLoS Biol. 2004 Nov 30;2(12):e422. doi: 10.1371/journal.pbio.0020422 (PMC532390; doi:10.1371/journal.pbio.0020422)
Supplement: Table S1 — Also available at http://genes.mit.edu/NielsenEtAl/. (4.3 MB ZIP). [file pbio.0020422.st001.zip › NielsenEtAl/html/691.html]

AN2934.1.NCU03281.1.MG00548.1.FG07059.1


```
 CLUSTAL W (1.82) Multiple Sequence Alignments - Introns Inserted


Sequence 1: MG00548.1	212 aa
Sequence 2: FG07059.1	161 aa
Sequence 3: NCU03281.1	200 aa
Sequence 4: AN2934.1	198 aa
Alignment Length: 217 aa
Number Identitical Residues: 62 aa
Alignment Score (without introns) 3369


MG00548.1 	MDAHAHHMMDHSGMDHG-GMDH---GGMDHGGHGGGGMDMDRCSMN0MLFTWSTKNLCIV
NCU03281.1	--------MDHSGHDHSTHMDHSGHGDMHSG-HPGMGEGAPRCSMN~MLFTWNTENLCIV
FG07059.1 	--------MDHSHMDHS-GMDH---GDMDHG-HGGGGMT-DMCSMN0MLFTWDTNNLCIV
AN2934.1  	--------MDMYHMGMD--MDH------SHHEHGDMDMGGNQCSMN0MLFTWSTKNLCIV
          	        **    . .  ***          * . .     **** *****.*:*****

MG00548.1 	FEQWHIRSTAGLIFSLLAVVAIGAGYEALRESIRRYEYHLNKKNEAVPL1EEQTK~SGVM
NCU03281.1	FRQWHIHSTFSLVVSLLAIVALAAGYEALREGIRQYEAWTNKRVETTPH1PREQ-~----
FG07059.1 	FRQWHVRSTTSLLFSLIAVIILAIGYEALRSVSRRYEQSLDNRVRSVPR1QSQ--~----
AN2934.1  	FSTWRVTGPLSLLGSLVVIVLLAAGYEGIRQVTRKYEVAHARRLSAFST~AVVGS1N---
          	*  *:: .. .*: **:.:: :. ***.:*.  *:**    .:  : .      . .   

MG00548.1 	NFYTMLSRTPAKIRRAEADDNVNENTPFVTPGQS-RPKVTREAHFVKAVLYGIQNFYAFM
NCU03281.1	--YRDDDDDDDVEQQLEDEERLAETTMPWLLGQN-VAAVTKRAHAIKSVLYGIQNFYAFM
FG07059.1 	-------------------------------GQ-----ADQRAHLIKAVLYALQNFYAFM
AN2934.1  	EFADEDVTGSGPGHESQSQAYVPNASSPLLVGSDNRRAMERRGKITMAALYGVQVFYSFF
          	.               . .    . :     *...     :..:   :.**.:* **:*:

MG00548.1 	IM2LIFMTYNGWVMLAVSFGAFVGYLLFGGSTPATKETACH
NCU03281.1	IM2LIFMTYNGWVMLACSLGAALGYFVFGSRTTATKETACH
FG07059.1 	LM2LVFMTYNGWVMVSVSLGAFLGYLFFGQRTSATKENACH
AN2934.1  	IM2LLFMTYNGFVMLAVAIGAFVGYLAFGENMSATKSVACH
          	:* *:******:**:: ::** :**: **   .***. ***
```
